# Coping with drought: stress and adaptive mechanisms, and management through cultural and molecular alternatives in cotton as vital constituents for plant stress resilience and fitness

**DOI:** 10.1186/s40659-018-0198-z

**Published:** 2018-11-14

**Authors:** Aziz Khan, Xudong Pan, Ullah Najeeb, Daniel Kean Yuen Tan, Shah Fahad, Rizwan Zahoor, Honghai Luo

**Affiliations:** 1 The Key Laboratory of Oasis Eco-agriculture, Xinjiang Production and Construction Group, Shihezi University, Shihezi, 832003 People’s Republic of China; 2 Key Laboratory of Plant Genetic and Breeding, College of Agriculture, Guangxi University, Nanning, 530005 People’s Republic of China; 3 Queensland Alliance for Agriculture and Food Innovation, Centre for Plant Science, The University of Queensland, Toowoomba, QLD 4350 Australia; 4 Plant Breeding Institute, Sydney Institute of Agriculture, School of Life and Environmental Faculty of Science, The University of Sydney, Sydney, NSW 2006 Australia; 5 Department of Plant Sciences and Technology, Huazhong Agriculture University, Wuhan, 430000 People’s Republic of China; 6 Department of Agronomy, The University of Swabi, Swabi, Pakistan; 7 College of Life Science, Linyi University, Linyi, 276000 Shandong China; 8 Key Laboratory of Crop Growth Regulation, Ministry of Agriculture, Nanjing Agricultural University, Nanjing, 210095 People’s Republic of China

**Keywords:** Drought, Leaf physiology, Antioxidant, CRISPR/Cas9, miRNAs, Climate change, Phytohormones, Zinc finger nuclease

## Abstract

Increased levels of greenhouse gases in the atmosphere and associated climatic variability is primarily responsible for inducing heat waves, flooding and drought stress. Among these, water scarcity is a major limitation to crop productivity. Water stress can severely reduce crop yield and both the severity and duration of the stress are critical. Water availability is a key driver for sustainable cotton production and its limitations can adversely affect physiological and biochemical processes of plants, leading towards lint yield reduction. Adaptation of crop husbandry techniques suitable for cotton crop requires a sound understanding of environmental factors, influencing cotton lint yield and fiber quality. Various defense mechanisms e.g. maintenance of membrane stability, carbon fixation rate, hormone regulation, generation of antioxidants and induction of stress proteins have been found play a vital role in plant survival under moisture stress. Plant molecular breeding plays a functional role to ascertain superior genes for important traits and can offer breeder ready markers for developing ideotypes. This review highlights drought-induced damage to cotton plants at structural, physiological and molecular levels. It also discusses the opportunities for increasing drought tolerance in cotton either through modern gene editing technology like clustered regularly interspaced short palindromic repeat (CRISPR/Cas9), zinc finger nuclease, molecular breeding as well as through crop management, such as use of appropriate fertilization, growth regulator application and soil amendments.

## Introduction

Recent climate change investigation has reported that global escalation of storms, flooding and other severe weather episodes with growing temperatures may ultimately disrupt crop production [1]. Global circulation models projected an increase of 4–5.8 °C in the surface air temperatures over the next few decades. From 1979 to 2003, an increase of 0.35 and 1.13 °C have already documented in the annual mean maximum and minimum temperatures respectively, at the International Rice Research Institute, Manila, Philippines [2–6]. These temperature increases have likely exposed crops globally to drought induced stress [7–15]. Tackled with shortage of water reserves, drought is the single most serious risk to world food safety. Drought severity is unpredictable as it depends on several factors, for instance rainfall amount and distribution, evaporative demands and moisture storing ability of soils [16–19]. Uncertainties in weather conditions can result in decreased rainfall coupled with increased evapotranspiration. These occurrences can lead to drought and substantial reductions in cotton yield. Over the las 50 years, drought stress alone was responsible for approximately 67% of the cotton lint yield losses in USA, one of the top cotton producing countries in the world [20].

Cotton crop is very sensitive to cold, soil salinity, heat and drought stress [21]. For instance, episodic drought events can cause severe lint yield penalty and may become a significant challenge for sustainable crop production [22]. On the contrary, even a small but adequately timed irrigation can significantly improve water-stressed crop [23, 24]. Drought-induced lint yield penalties in cotton may vary from 50 to 73% [25]. In dry the regions, accounting that 30–60% of total irrigation water supplied to the soil is lost through evaporation which may cause drought [26]. Crops producing areas are already facing a continuous decline of irrigation water [27], and hence, there is a need to establish and design policies to protect crops from extreme weather events [28]. One of the most commonly used strategy, is breeding of stress-tolerant crop for any water-scarce based calamity.

Yield improvement and yield stability under both normal and moisture stress environment is essential for cotton crops. Tolerance to drought stress in cotton is a complex trait that depends on various environmental and physiological factors. Therefore, a sound understanding of the plant morpho-physiological, molecular and biochemical mechanisms, responses to water deficit may provide a means to identify and confer tolerance in terms of agronomic, molecular, and genetic aspects. The adaptive strategies used by drought tolerant plants are of major importance for improving performance of cotton crop under erratic water deficit conditions.

For instance, improvements in production systems and breeding programs have substantially increased cotton lint yield. These published literature will enable the development of crop plants better able to tolerate and thrive under future climatic conditions and so maintain production potential. To our knowledge, no such comprehensive and accumulative data are available to elucidate biochemical, morphological, physiological and molecular adaptive mechanisms of cotton to harsh environment, particularly drought.

## Structural and physiological responses to drought

Drought results in a wide range of variations at the developmental and functional level of cotton plants. For example, drought severely impedes various physiological processes, which regulate lint production and fiber quality [29].

Drought resistance mechanisms in plants are composed of four categories: recovery, avoidance, tolerance and drought escape [30]. Water stress avoidance is the sustaining of important physiological processes such as stomatal regulation, when exposed to mild drought. Drought tolerance is the ability of flora to endure severe dehydration via osmotic adjustment and osmo-protectants [31]. Plants are evolved to regulate growth period to avoid moisture stress; termed as drought escape [32]. Drought recovery is the ability of plants to continue growth after drought injury. In cotton, biochemical, physiological and molecular strategies against drought stress are reviewed in the proceeding sections.

## Root growth

Plant roots are crucial for sensing and responding to various external environmental stimuli due to direct contact with soil water and nutrients. Due to difficulty in collecting root structural configuration from dry soils, limited data are available on modification in root systems under drought and most of the studies are conducted on cereal crops. Plant roots respond to the variation in surface soil moisture e.g. water deficit in the upper soil profile leads to deeper root penetration, while excess water in the upper layer reduces root penetration [33], i.e. up to 3 m.

Root growth rates are commonly employed for estimating crop yield losses in cotton crop. Insufficient soil moisture restricts root growth and development and consequently impairs functioning of the aerial parts [33]. Water deficit in the upper soil profile leads to deeper root penetration for greater exploration of moisture and nutrients, while excess of water in the upper layer causes reduced root penetration [33]. Drought reduces above-ground biomass accumulation by decreasing root volume density, root mass density and root length density [34]. These root traits are crucial in the process of tolerance to drought; however, traits such as hydraulic conductance and plant allometry are of great interest to scientists. Rooting system with large number of short and slender lateral roots permits a larger root surface sorption zone than the scattered-type root system in acquiring oxygen and nutrients from soil [22]. Fine root system drives soil processes like carbon cycling and sequestration, nutrient fluxes, structural stabilization and the activity of soil microorganisms [35]. In soil, higher root length, and proliferation in the soil are desirable traits for drought adaptation. However, plant root growth and penetration depend on external oxygen partial pressure in the root zone [36]. Mild drought stress during the initial stage may enhance root elongation but root morphological and physiological activities are seriously hampered under long term water stress hampers [37]. In conclusion, deeper root penetration allows the plant to get greater exploration of deeper soil for water and nutrients. Therefore, it is essential to enhance vertical root distribution to enhance crop growth and development under drought stress.

## Cotton lint yield

Lint yield in cotton crops is a complex integration of the various physiological processes; most of which are adversely impacted by water stress. Due to indeterminate growth habit, production of new nodes in a cotton plant depends on water availability. The adverse effects of moisture stress on the yield are associated with the duration and severity of the stress and plant growth stage. Terminal drought substantially limits cotton yield production by inhibiting carbon assimilation, and biomass accumulation [38]. Inhibited carbohydrate production coupled with depletion of stored reserves (i.e. starch) due to continuous respiration [39] reduce translocation of assimilates to reproductive organs [40]. This consequently induces abscission of reproductive structures and boll size reduction [41]. Accelerated abscission of fruits and leaves in drought-stressed cotton crop could be associated with final yield reduction [42]. In brief, cotton yield reduction is directly associated with plant morphological and physiological processes under drought stress.

## Fiber quality

Fiber quality is a main aim of cotton breeders both because fiber traits directly affect lint yield and improvement in spinning technology has an increased demand for high-grade fiber [43]. Fiber quality is the combination of fiber length, fiber fineness (cell wall thickness), fiber strength, fiber elasticity, neps (small nodules on the fiber), short fiber index, uniformity index, spinning consistency (suitability of fibers for yarn-spinning), color grade, and reflectance (brightness of fibers) [44]. Fiber quality traits are quantitative and controlled by multiple genes with major and minor phenotypic effects [45]. Water supply during fiber cell development has a direct impact on lint quality [46]. As drought tolerance in plants is a complex phenomenon, associated with a variety of morphological and physiological traits [47], breeding for improved fiber quality traits under moisture stress is cumbersome [48]. Hence, the identification of stable quantitative trait loci (QTL) for irrigated and water deficit environment could facilitate molecular breeding of cotton genotypes with both improved fiber quality and yield attributes. QTL, genetic diversity and structure analysis, require the availability of abundant DNA markers which are continually being developed for the cotton genome [49]. In upland cotton, several QTL analyses have focused on lint yield traits [50, 51], but less attention have been paid to identify QTLs for fiber quality under drought [52]. Saranga et al. [53], used inter-specific F2 and F3 cotton plants derived from a cross between inbred lines of *G. hirsutum* cv Siv’on and *G. barbadense* cv F-177. QTLs (13 and 33) under well-watered and water deficit conditions and reported for 16 QTL trait including plant productivity, physiology and fiber quality. Paterson et al. [54] identified 79 QTLs allied with fiber quality traits in F2 and F3 generations derived from *G. hirsutum* cv Siv’on and *G. barbadense* cv F-177 under irrigated and deficit water conditions. Seventeen of the identified 79 QTLs were specific to moisture stress conditions, whereas only two were specific to well-watered conditions. Saeed et al. [52] mapped physiological, yield and plant structure traits in an F2 population generated from a cross between *G. hirsutum* cv. FH-901 (drought sensitive) and *G. hirsutum* cv. RH-510 (drought tolerant). A total of seven QTLs were detected of which three and two QTLs were specific to water-limited and well-watered conditions, respectively. Such QTL analysis of germplasm panels, which contain *G. hirsutum* lines with diverse genetic information have the capacity to detect a broader array of useful alleles. In the present study, 177 simple sequence repeat (SSR) markers were used to detect significant quantitative trait loci (QTLs) linked to 11 fiber quality and plant structural traits in a panel of 99 upland cotton genotypes. In another study, fiber quality and plant structural traits were tested under well-watered and water deficit conditions. Analysis of GLM showed that a total of 74 and 70 QTLs under well-watered and limited water conditions were identified, respectively. MLM identified 7 and 23 QTLs under well-water and water deficit, respectively [44].

For instance, for traits of important interest in cotton fibre, efforts have been made to detect the specific fiber associated gene and their functions for improved fiber quality i.e. E6 [55], GhExp1 [56], GhSusA1 [57], PIP2s [58] and GA20ox [59]. Cotton functional genomics promise to enhance the understanding of fundamental plant biology to systematically exploit genetic resources for improvement of cotton fiber quality. However, determining the functions of cotton genes is cumbersome, which has not been fully assessed a rapid pace [60]. Actin cytoskeleton [61], polysaccharide biosynthesis, signal transduction and protein translocation [62] associated genes are expressed in different fiber developmental pathways. Among these genes, few are predominantly expressed during fiber formation [63], secondary cell wall biosynthesis [64], and fiber elongation [65]. Currently, a cotton protodermal factor 1 gene (GbPDF1) was expressed at fiber initiation stage through HDZIP2ATATHB2 core cis-element [66]. While, alpha-expansins (GhExp1) gene had been expressed in developing fibers and encodes a cell wall protein and controls cell wall loosening [56]. Ruan et al. [67] showed that antisense suppression of a sucrose synthase (SuSy) gene interrupted the fiber elongation and signified the contribution of SuSy in osmosis regulation. Conversely, proline-rich proteins coding gene (GhPRP5) performed as a negative regulator during fiber development [68]. Cellulose synthesis is a central event in fiber cells development during the secondary cell wall biosynthesis. Many studies have been done to investigate that how cotton fiber regulates and supports the strong irreversible carbon sink characterized by secondary wall cellulose synthesis [64]. Subsequently, a new Sus isoform (SusC) was discovered, which was up-regulated during secondary wall cellulose formation in the fiber [64]. At fiber maturity, most of the expressed genes were linked to cellular respiration [69]. Many genes encoding transcription factors i.e. MYB, C2H2, bHLH, WRKY and HD-ZIP families have also been expressed during the fiber developmental stage. Past studies indicated that MYB-related genes showed high expression during fiber development in upland cotton [70]. Expression studies of six MYB-associated genes revealed that GhMYB6 has high expression in fiber [71], while R2R3 MYB-like transcription factor encoding gene “GhMYB109” was expressed during fiber elongation and initiation [72]. In the cotton ovule, at fiber initiation the RAD-like GbRL1 is highly expressed [73].

Identification of markers connected to loci for fiber quality under moisture stress can have useful effects in genetic adaptability required to generate necessary fiber under limited water conditions. Numerous gene expression investigations had been performed to understand on cotton fiber development which present some issues. Firstly, majority of differentially expressed genes known by the comparative analysis are linked to difference between species instead of allied with fiber characteristics. Secondly, the application of the protein coding gene sequences from *G. raimondii* and *G. arboreum* may not be accurate enough for gene annotation in tetraploid cotton. Thirdly, it is unfamiliar if any of the expressed genes identified from earlier studies had sequence changes between a cotton fiber mutant and its wild-type. Herein, only the differentially expressed genes having sequence variations and co-localization with target fiber characteristics are potential candidates for innovative cotton research.

## Photosynthesis

**Fig. 1:**
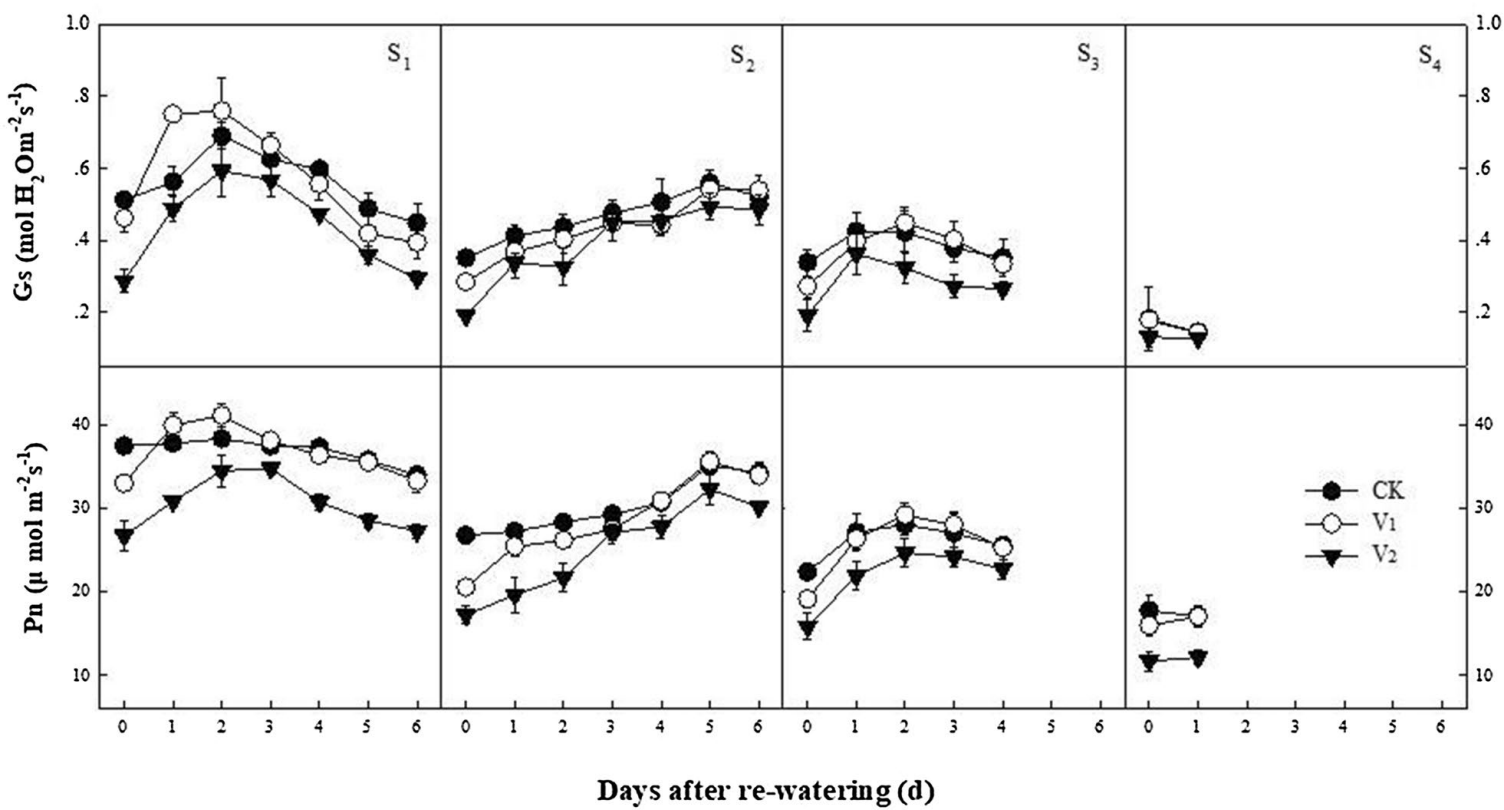
Changes in stomatal conductance (gs) and net photosynthetic rate (*Pn*) of cotton leaves in response to drought stress and recovery. The periods are full squaring to flowering (S1), first flowering to full flowering (S2), full flowering to full boll setting (S3) and full boll setting to boll opening stage (S4), respectively. The water treatments were mild stress or 50–55% of maximum soil water (V1), moderate stressor 40–45% of maximum soil water (V2), and a well-watered check

Photosynthesis is the main driver for crop productivity, which is negatively influenced by water deficit conditions. Stomata closing in response to moisture stress results in a reduction in leaf photosynthetic capacity resulting in chloroplast dehydration and decreased CO_2_ diffusion into the leaf (Fig. 1). For instance, mild moisture stress stimulates stomata closure to reduce water loss by regulating transpiration. This reduces stomatal conductance and limits intercellular CO_2_ concentration [74]. Under severe drought, reduced stomatal conductance and metabolic (non-stomatal) damage like limited carboxylation becomes major limitations to photosynthesis [75]. Similarly, stomatal conductance is not constantly allied with photosynthesis, although it needs to be investigated [76, 77]. Drought can reduce photosynthesis as well as severely affect transpiration rate and the process depends on the intensity of drought and plant developmental stage [78]. Up to 66% reduction in photosynthesis was noted in mature cotton leaves compared with younger leaves under water deficit conditions [79].

## Stomata regulation

In plants, the main role of stomata is to regulate water loss through transpiration. Under moisture stress, internal moisture preservation and quick stomatal closure are vital for plant withstand to water deficit conditions. Water loss from cotton leaves is a key phenomenon under water deficit conditions but plants use morphological adaptation to survive under drought stress i.e. leaves wilting and rolling leading to less radiation interception, and ultimately decreased water loss [30]. Plants usually show numerous xeromorphic traits and structures that induce drought resistance, i.e. a thick cuticle epidermis, thicker and tiny leaves, smaller and denser stomata, palisade tissues more epidermal trichomes, and a well-structured vascular bundle sheath [80]. Stomatal regulation plays an imperative role in leaf gas exchange between the intracellular cavity of the leaf and external environment. Plant leaves dissipate heat energy through three means. These mechanisms are re-radiation, sensible heat loss (conduction and convection) and transpiration. Of these, transpiration is the most important mechanism that sanctions plants to harvest energy and sustain cellular functions. As 90% of water loss from plants occurs though stomata openings via transpiration [81], stomatal regulation plays a key role in maintaining water and nutrient supplies for essential physiological process. Under high transpiration, stomata closure is the initial step to decrease water loss under drought conditions in cotton crop. Ray and Sinclair [82] reported that among the eight corn (*Zea mays* L.) hybrids there were statistical differences in the fraction of transpirable soil water at which the stomata began to close during a drying cycle. Hence, stomatal conductance would be a possible indicator for inducing drought tolerance, although a negative correlation is associated between drought resistance and stomata conductance in cotton.

## Osmotic adjustment

**Fig. 2:**
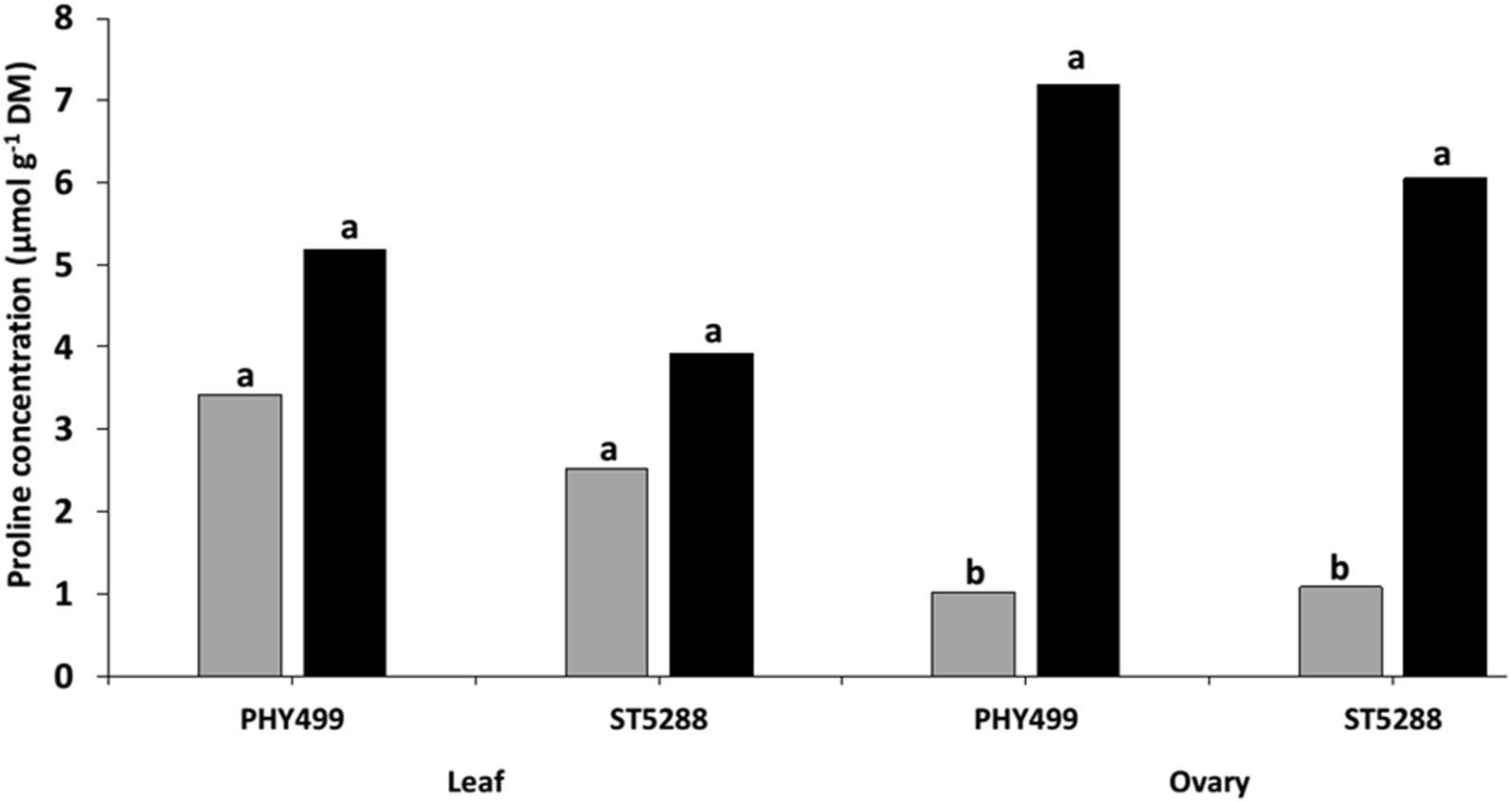
Proline concentration (μmol g^−1^ DM) in the leaves and ovaries of two cotton cultivars. The water treatments were water stress (black bars) and well–watered (gray bars). Different letters indicate significant differences (P ≤ 0.05) [84]

Osmotic adjustment is an acclimation strategy to sustain higher cellular turgor potential and water retention against moisture stress. In other crop species, osmotic adjustment of leaf is strongly correlated with drought resistance. In response to water stress, osmotic adjustment take place in plant cells via increased of compatible solutes in the cytosol. This reduces the osmotic potential of the cell to sustain cell turgor and development. Compatible solutes like proline, sorbitol, and glycinebetaine, and are more soluble and do not interfere with cell metabolism even at higher concentration [83]. In plants, proline is a common compatible solute under drought stress [83]. However, proline accumulation in droughted plants vary and depends on cultivar and growth stage, (e.g., in cotton ovaries proline accumulation was higher than in the leaf) (Fig. 2). Pilon et al. [84] indicated that osmotic adjustment in cotton during reproductive stages may be higher than during vegetative stages and possibly tissue dependent.

Compatible solutes protect proteins and membranes from the injury occasioned by elevated concentrations of inorganic ions and oxidative damage under water deficit [85] and salinity [86]. Foliar applied proline and glycinebetaine could be an effective strategy for inducing drought tolerance in cotton crops [87]. In cotton plants more glycinebetaine accumulation exhibited more drought tolerance. Thus, promoting physiological process e.g. leaf photosynthetic capacity, relative water content, enhanced osmotic adjustment and low lipid stability through transgenic or non-transgenic techniques may improve crop performance under drought [88]. For example, constitutive expression of a mustard annexin gene, *AnnBj*, increased proline and sucrose content in cotton resulted in greater drought tolerance [89]. Furthermore, overexpression of *GhAnn1,* annexin genein cotton, induced drought and salt tolerance by improving superoxide dismutase, activity raised proline concentration and increased soluble sugars [90]. Further studies are needed on osmotic adjustment in reproductive organs to fully understand this mechanism in cotton plants under drought.

## Biochemical and molecular mechanisms of drought tolerance

Plants avoid a range of external stresses through morphological adaptation. The mechanism of drought tolerance is linked to several biochemical, morpho-physiological, and molecular processes. These processes are intensely regulated by the hormonal interplay within the plant body.

## Abscisic acid (ABA)

Abscisic acid (ABA) is a natural plant stress hormone and controls; stress responses, growth, and reproduction in crop plants. Osmotic stress in plants is related with a degree of drought and low water availability [91], which induces ABA synthesis, and adaptive mechanisms [92]. After stress signals reception by the plasma membrane, abscisic acid synthesis is initiated and occurs in the plastids with the exclusion of xanthoxin transformation to ABA. This occurs in the cytoplasm [93]. ABA is generally synthesized in roots and transported to upper parts of the plant via vascular tissues [94]. In cotton, perception and signal transduction of ABA are facilitated either by ABA-dependent or ABA-independent passageway, where the former is key player in the expression of stress-responsive gene during numerous stresses, particularly under osmotic stress. Numerous receptors have been recognized in plasma membrane, cytosol, chloroplast envelope and nucleus. Under non-stress environment, plants show low ABA content and sucrose non-fermenting 1-linked protein kinase 2 (SnRK2) proteins action is subdued via protein phosphatase 2C (PP2C), which results in dephosphorylation. In cotton plants, ABA improves drought tolerance by regulation of stress-associated gene. Overexpression of ABA-induced cotton gene *GhCBF3* in *Arabidopsis* led to drought resistance in transgenic lines through maintaining higher relative water levels, chlorophyll and proline content than wild type [95]. Compared with wild type transgenic line, *AREB1* and *AREB2* show higher expression levels, while lower stomatal aperture upon treated with ABA. Suggesting that, *GhCBF3* can improve drought resistance through ABA signaling pathway.

## Jasmonic acid (JA)

Jasmonic acid (JA) is regarded plant phytohormone and its active derivatives termed jasmonates. It plays a key role in combating several biotic and abiotic stresses. Furthermore, better root structure, tendril coiling, pollen production and fruit ripening are associated with JA [96]. Studies reported that exogenously applied jasmonates increases plant performance under arid environments [97] and regulate stomatal dynamics [98]. Jasmonic acid signaling pathway and biosynthesis have been widely studied [99]. The jasmonate-zim domain (JAZ) repressor protein plays significant roles in the JA signaling pathway which perform as a switch for JA signaling. Under non-stress environment and absence of JA, jasmonate-insensitive/jasmonate-zim (JAI3/JAZ) proteins link to numerous transcription aspects including myelocytomatosis (MYC2) and suppress their activity. Nevertheless, under deficit water, when JA and its derivatives are present, degradation of JAZ proteins happens as depicted above, causing active transcription factors i.e. MYC2, that up-regulate genes associated with stress tolerance [100]. Usually, plant hormones do not perform in single pathways, but somewhat depended on each other at various phases to regulate ambient and developmental pathways. In plants, signal transduction arises and can organize numerous developments to respond to harsh environment in a complicated way [98].

## Reactive oxygen species (ROS)

Fractional reduction of atmospheric O_2_ causes the generation of reactive oxygen species (ROS) also called active reactive oxygen intermediates (ROI). Cellular ROS is composed of four categories i.e. the hydroxyl radical (HO·), superoxide anion radical (O_2_^−^), hydrogen peroxide (H_2_O_2_), and singlet oxygen (^1^O_2_). HO· and ^1^O_2_ are relatively more reactive and can oxidize DNA, and RNA, lipids and proteins, ultimately causes cell death [101]. Sub-cellular sites, i.e. cell wall, chloroplast, nucleus, mitochondria and plasma membrane, induces ROS production [102]. Production of these ROS raises under drought e.g. a reduction in CO_2_ fixation results in diminished NADP^+^ redevelopment during the Calvin cycle. This decreases photosynthetic electron transport chain the activity. Furthermore, too much electrons leakage to O_2_ by the Mehler reaction in drought-treated cells can also improve ROS production during photosynthesis [103]. The Mehler reaction lessens O_2_ to O^2−^ by donation of an electron in photosystem I. O^2−^ can be transformed to hydrogen peroxide by superoxide dismutase which can be further transformed to water by ascorbate peroxidase [104]. Nevertheless, it is hard to assess the levels of ROS generated during the Mehler reaction relative to those produced via photorespiration. Moisture stress also increases the photorespiratory pathway, principally when RuBP oxygenation is high owing to partial CO_2_ fixation. Approximately 70% of the total H_2_O_2_ production under moisture stress takes place via photorespiration [105].

Plants have complex scavenging mechanisms and controlling pathways to screen ROS redox homeostasis to avoid additional ROS in cells. In cotton plants, changes in antioxidant enzyme metabolism can affect drought resistance. The antioxidant systems have been developed by plants to continue their growth. This system is composed of enzymatic and non-enzymatic complements. These enzymes are superoxide dismutase, ascorbate peroxidase, guaiacol peroxidas, monodehydroascorbate reductase, catalas, dehydroascorbate reductase and glutathione reductase. Reduced ascorbic acid (AA), flavonoids, carotenoids, proline, glutathione and (GSH), α-tocopherol are the non-enzymatic constituents. These two constituents act together to scavenge ROS [106, 107]. Ascorbate peroxidase, combine with NADH MDAR, and GR, detoxify H_2_O_2_ through the Halliwell–Asada pathway [107]. Ascorbate reduces MDHA to MDHAR. Nevertheless, 2 molecules of MDHA can be non-enzymatically transformed to MDHA and dehydroascorbate, which is further reduced to ascorbate through the NADH and GR cycle [108]. Glutathione (GSH) is reduced by GR oxidation at the presence of NADPH. Glutathione reductase activity increases under moisture stress to retain oxidized and reduced glutathione ratios at certain levels [109]. The equilibrium between antioxidative enzyme activities and ROS production decides, if oxidative signaling and/or loss occur [110]. The antioxidative ability of different cotton cultivars regulates the resistance potential to dry conditions. In cotton, moisture stress induces ROS production, but in contrast, the APX and GR activities can also improve and sustain the ROS scavenging process [111]. Nutrient (Zn) application have been found to minimize polyethylene glycol (PEG)-induced oxidative damages in cotton. This increases CAT, APX, SOD, activities and non-enzymatic antioxidants content [112]. Zhang et al. [111] found in a drought-tolerant (CCRI-60) cotton line has led to increased GR activity and improved proline level. Compared with the sensitive (CCRI-27) CCRI-60 had potential to scavenge free radicals and protect the plants from harsh conditions. As a result, indicating improved growth and induced tolerance in response to drought stress. Down-regulation of *GbMYB5* in *G. barbadense* led to reduced antioxidant enzyme activities include CAT, peroxidase (POD), SOD, and glutathione S-transferase (GST), and enhanced oxidative stress when exposed to moisture stress [113]. However, further investigations are needed to identify genes involved in the antioxidant enzyme-related pathways in drought resistant cotton cultivars. Additionally, application of Zn and K supplies can also improve the antioxidant system of cotton plants [107–114].

## Strategies to improve drought tolerance

Globally, numerous management strategies are implemented for better crop production under stressful environments. The combined application of various management options is critical due to agricultural, financial, social and ecological limitations. Among these, nutrients management is regarded a quick and more effective better strategy to tackle abiotic stresses. Incremental transformations in cotton productivity are important, particularly in arid regions due to limited soil water availability. In this context, cotton crop that demand a lesser amount of water but produce optimal yields and good quality fiber will be more necessary. Despite the traditional breeding programs, improvement in biotechnology can harvest desirable cotton crops that produces optimal yields in the current and future harsh environmental conditions. Exogenous use of growth regulators, soil amendments, specific osmoprotectants, and essential nutrients can enhance drought resistance in vulnerable plants.

## Exogenous application of substances

### Application of hydrogels

Hydrogels are super absorbent polymers and possess the potential to retain substantial quantity of water. Utilization of these substances in many industrial and environmental zone may be possible [115]. In agriculture, water retention capacity of soil can be enhanced by the addition of hydrogels. Cellulose, pectin, chitin and carboxymethyl cellulose (CMC), are the natural macro-molecules having high potential to absorb water to form hydrogels. They swell quickly and hold enormous amount of water in three-dimensional structure, when placed in water [116]. In controlled release systems, the utilization of hydrogels since the availability of suitable nutrients in soil is an aspect for crops productivity [117]. Furthermore, existence of hydrogels in the soil increases water availability and decreases nutrients loss by percolation and leaching. Enhancement of soil aeration and drainage, and a faster rate of plant root and shoot growths [107]. In conclusions, use of hydrogels enhances soil water retention capacity and water uptake. Hydrogels can improve plants performance by enhancing soil permeability, infiltration rates, reducing irrigation frequency, decreasing soil erosion and lessen water loss.

### Appropriate and adequate fertilization

Maintenance of adequate potassium (K) nutrition to plants has been found critical to mitigate drought stress. Potassium is the most important plant macro-nutrient with regard to cotton water relations, and it influences plant biochemical and physiological processes regulating to development and metabolism [118]. Compared to control conditions, K fertilization can significantly improve cotton yield and yield contributors under stress (Table 1).

**Table 1:** Effect of short-duration drought stress and recovery after re-watering at flowering stage on seed cotton yield (SCY) and its components in two different cotton cultivars under different K rates

Cultivar	Water regime	K level	Number of bolls/plant	Boll weight (g)	SCY/plant (g)
Siza 3	Control	0	14.6c	4.9c	71.41c
		150	16.8b	5.5a	93.20b
		300	18.2a	5.7a	103.15a
	Stress	0	7.6f	4.5d	35.94e
		150	11.3e	5.2b	58.68d
		300	12.9d	5.5a	67.98c
Simian 3	Control	0	16.1b	4.6cd	74.25c
		150	17.6a	4.9b	86.22b
		300	18.6a	5.2a	95.86a
	Stress	0	8.8e	4.2e	38.09f
		150	11.4d	4.5d	50.34e
		300	13.0c	4.7c	58.55d

For each cultivar, values followed by a different letter within the same column are significantly different at *P* ≤ 0.05 probability level. Each value represents the mean of three replications [114]

**Fig. 3:**
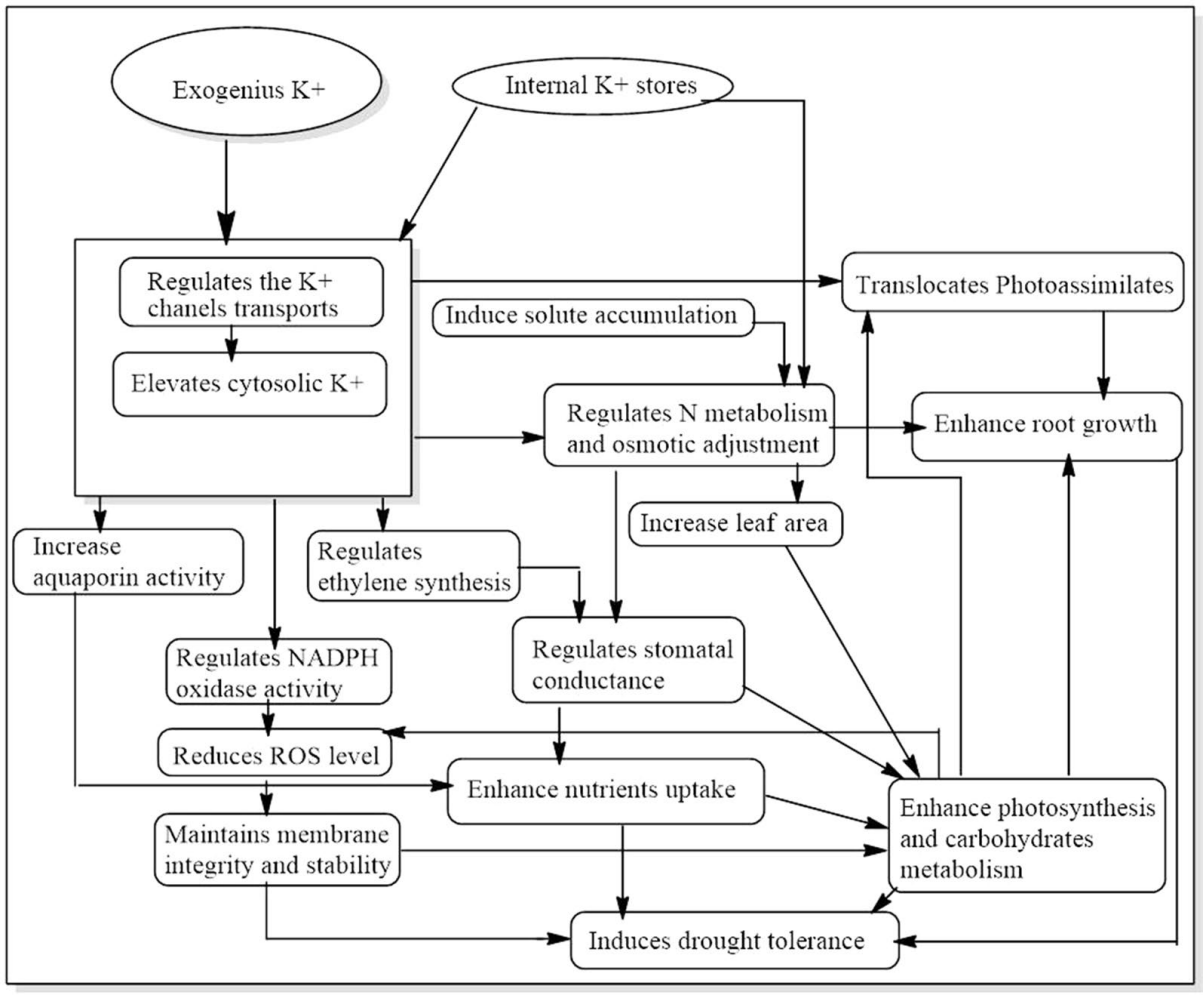
The relationship between potassium supply and morpho-physiological characteristics in response to water deficit conditions in cotton crops

ROS generation in drought stressed crops may further be increased when combined with K deficiency [119]. CO_2_ fixation in K-stressed plants greatly restricted by impairment in stomata regulation when subjected to drought. Under drought, in plant cells increasing extra-chloro-plastic K^+^ concentrations with surplus application of K^+^ could prevent photosynthesis inhibition under moisture stress [120]. An adaptive K demand for drought subjected plants may be associated to the role of K in enhancing photosynthetic CO_2_ fixation and transport of photosynthates to sink. By this way inhibiting the transfer of photosynthetic electrons to O_2_ resulting in low ROS production [119]. A recent study demonstrated that K fertilizer application induced drought tolerance in cotton plant by improving leaf photosynthetic capacity, biomass accumulation and enhanced down-stream carbohydrate metabolism [120]. In summary, an adequate K supply can increase cell membrane stability, leaf area, root growth, and final biomass for drought stressed plants and also improves water uptake. This indicate that, appropriated K nutrition is important for plant osmotic adjustment and alleviating ROS impairment. The potential roles of K in plants exposed to drought stress are presented in (Fig. 3).

### Growth regulators

Both, natural and synthetic plant growth regulators can reduce the adverse effects of deteriorated environmental conditions on plant development. Foliar application of growth regulators and osmoprotectants can also improve drought resistance in cotton. Exogenous application of osmoprotectants and plant hormones such as salicylic acid (SA), proline, ABA, glycinebetaine, polyamines and gibberellic acid (GA_3_) have been found to induce moisture stress tolerance. In plants, these hormones, elevated osmotic adjustment to increase turgor pressure and enhance addition of antioxidants to detoxify ROS; sustaining the integrity of membrane structures and macromolecules under moisture stress environment [121]. Glycine betaine and proline have been reported effective in minimizing the negative impacts of water stress on cotton. Similarly, exogenous GA use can enhance leaf photosynthetic capacity, stomata conductance and transpiration rate of cotton crop [122].

### Molecular basis of drought tolerance

Tolerance to water stress is a quantitatively controlled trait in plants. Drought changes the expression of genes, regulating water transport, oxidative damage, osmotic balance and damage repair mechanism. Recently developed molecular tools e.g. RNA-Seq and bio-informatic have accelerated the discovery of stress responsive genes in many plant species. For example, 33 QTLs have been identified in F3 plants originated from a cross between *G. barbadense* and *G. hirsutum* for water deficit conditions, including 5, 11 and 17 QTLs for physiological traits, plant productivity and fiber quality, respectively [123]. Most of the identified QTLs for drought tolerance were located on c2, 6 and 14 chromosomes [53]. Levi et al. [124] used marker-assisted selection for generating near-isogenic lines with greater drought tolerance and yield potential from the *G. barbadense* and *G. hirsutum* hybrids. Further, the *G. hirsutum* plants displayed greater levels of metabolites and more stable photosynthesis compared with *G. barbadense* under drought and well water conditions [124]. Similarly, QTLs related to osmotic potential (on chromosome c1, c2, 6 and 25), chlorophyll (on c2 and c14) and leaf morphology have been identified in drought tolerant cotton genotypes. In addition, 15 markers were found related to drought tolerance in 323 *G. hirsutum* genotypes with the help of microsatellite markers. Out of these, 12 markers showed negative and the remaining showed positive allele effects for drought tolerance [125]. Likewise, Zheng et al. [123] identified 11 physiological and morphological marker traits linked with drought tolerance in the field-grown cotton crop, while 67 and 35 QTLs were expressed under water drought and non-drought environment, respectively. These QTLs were mainly located on chromosome c16, c9 and c2.

### Role of miRNAs in drought stress alleviation

**Fig. 4:**
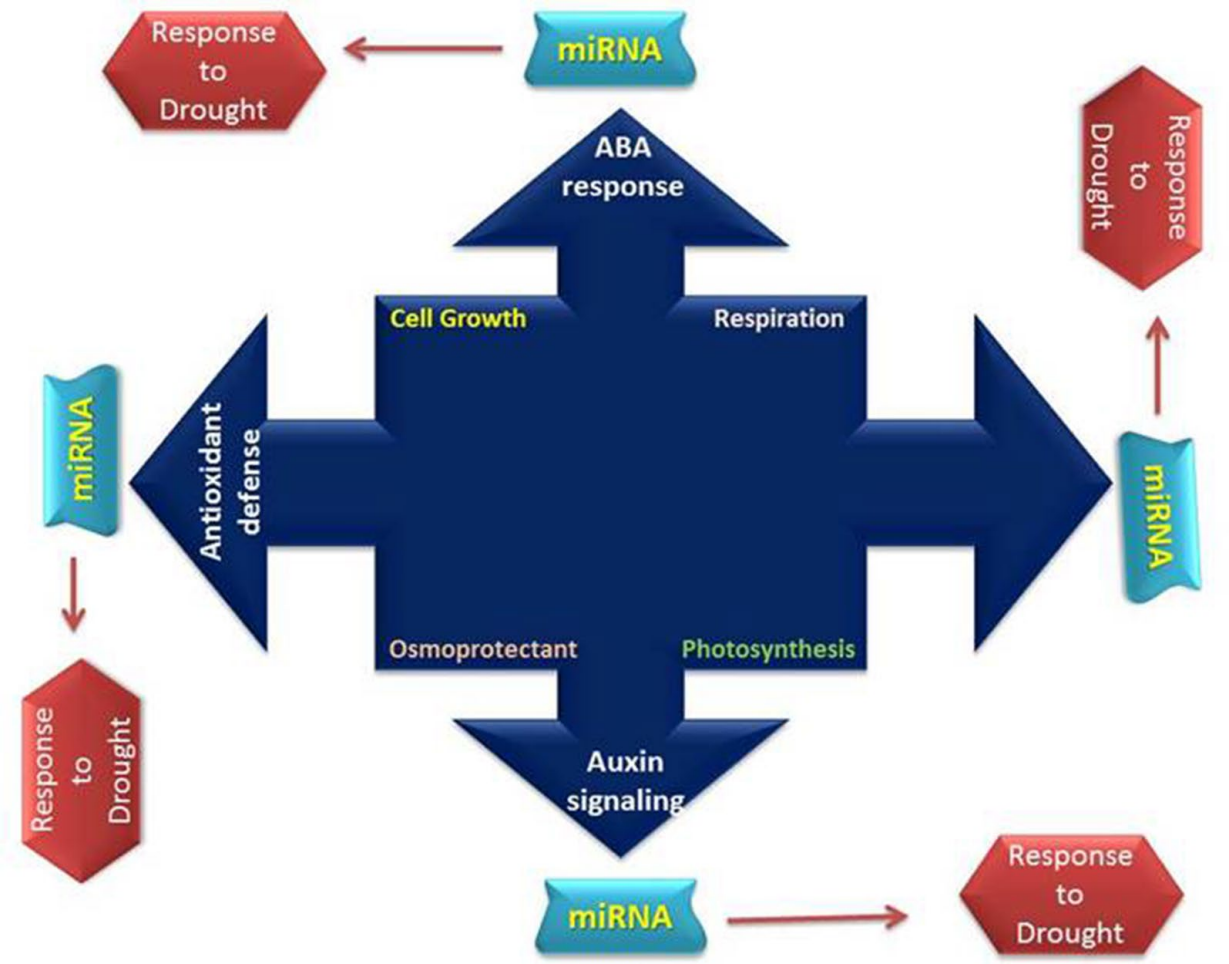
Different cellular processes in association with miRNAs for drought tolerance in plants

MicroRNAs (miRNAs), a class of endogenous non-coding small RNAs molecules, play an imperative role in response to several abiotic stresses [126]. For instance, hormone mediated signaling cross talk in crops was involved in combating drought stress, i.e. abscisic, ethylene and salicylic acid [127]. In plant, gene expression and hormonal regulation is an effective strategy to combat drought [128] which are in turn are controlled by miRNAs. Drought stress responsive miRNAs are shown to participate in various crops species like *Arabidopsis* [129], rice [130], cotton [131] and *Brassica napus* [132]. The role of miRNAs under drought stress have been presented (Fig. 4). In the current agricultural systems, scientists are paying more attention to improve lint yield and quality and the mechanisms of fiber development and adaptation of drought [133]. Exposure to long term water deficit conditions can cause in serious metabolic disorders in cotton plants leading to tissue dehydration, ionic toxicity and nutritional imbalance [134]. In cotton over-expression of GhCIPK6 gene improved drought resistance. This indicates that it could be a helpful to combat moisture stress in cotton [135]. Transgenic tobacco overexpression of the C mitogen-activated protein kinase gene (GhMPK2) in cotton group resulted in lower water with greater resistance to drought. This indicates that GhMPK2 might be positively adjusts drought resistance in cotton [136]. A sequence of cotton miRNAs is associated with genes, such as miR172, miR6158, miR396, miR164, ghr-n56, ghr-n59, ghr-n24 and miR1520. Interestingly, 163 cotton miRNAs were explored to target 210 genes associated with fiber development [131]. In brief, miRNAs are novel tool to improve plant performance under harsh environmental conditions. More studies on the function and expression of these miRNAs will be needed to explain their regulatory role in inducing tolerance to harsh environments.

### Transgenic approach

At molecular level, plants respond to abiotic stresses by altering expression of genes that in turn regulate protein synthesis and biological functions [137]. Regulation of these genes imparted stress response is an essential factor in plants that enhance plants growth and development under abiotic stresses. Various pathways associated with drought tolerance have been identified in transgenic cotton cultivars under controlled and field (Table 2). Out of thousands of identified genes, only fewer genes were associated with drought tolerance. Various genes, regulating response of Pima cotton (*G. barbadense*) to stressed environments e.g. drought, salt, heat, cold, and phosphorus deficiency have been identified using normalized cDNA libraries [138]. These desired traits may be transferred to upland cotton (*G. hirsutism*) through intraspecific breeding or genetic engineering techniques [139]. For example, transfer of genes from *Thellungiella halophile* overexpressing *TsVP*, an H^+^-PPase into cotton genotypes enhanced shoot and root growth than wild type. This improved performance of transgenic lines was the result of higher leaf chlorophyll level; photosynthesis efficiency, water content and cellular thermos-stability. Improved root structure and lower solute potential of transgenic plants enabled transgenic cotton to produce 51% more seed cotton than the wild type cotton [140]. Transfer of ScALDH*21* gene from *Syntrichia carninervis* induced drought tolerance in cultivated cotton [141]. At natural environment, transgenic lines produced greater biomass, bigger bolls and fiber yield relative to the wild type under drought stress. This superior performance of transgenic cotton was achieved through improved physiological traits e.g. higher proline and soluble sugars, photosynthesis and lower lipid peroxidation. These discoveries have encouraged scientists to engineer water deficit resistance crops (cotton) through genetic engineering technology. As a result, various desired genes have been transferred into cotton plants. However, field validation of these transgenic plants is still needed as most of these experiments have been performed under controlled environments and did not produce significant results in the field.

**Table 2:** Successful stories of GM plants against drought stress

Environmental condition	Stress type	Beneficial features for drought tolerance	Yield	References
Greenhouse and field	Drought	Improved water use efficiency (WUE), photosynthesis, root system and osmotic adjustment and scavenging ROS	NA	[142]
Laboratory and green house	Drought and heat	Enhanced protection of photosynthesis, seedlings and leaf viability	NA	[137]
Laboratory, greenhouse and field	Drought and salt	Increased proton pump activity of the vacuolar pyrophosphatase, auxin polar transport stimulation lead to root development	Increased 20%	[143]
Laboratory and greenhouse		High chlorophyll content, improved photosynthesis, higher relative water content and less cell membrane damage	Increased 40%	[140]
Laboratory, greenhouse and field	Drought	Increased production of ABA and proline content	NA	[144]
Green house		Enhanced proline content and root development, while transpiration rate decreased	131% more bolls	[145]
Green house and field	Drought and salt	Enhanced sequestration of ions and sugars into vacuole, reduced water potential, and enhanced root biomass	20% increased	[146]
Greenhouse	Drought	Higher relative water content and proline level while reduced H_2_O_2_, lipid peroxidation and electrolyte leakage	57.6%, more bolls	[147]
Greenhouse	Drought	Improved photosynthesis, roots and shoots, higher relative water content and less cell membrane damage	51% higher	[88]
Greenhouse	Drought	Increased photosynthesis, higher relative water content, better osmotic adjustment, less ion leakage and lipid membrane peroxidation	3–12% more	[148]
Greenhouse	Drought	Higher photosynthesis, delayed leaf senescence	NA	[149]
Greenhouse	Drought and salt	Longer roots, higher chlorophyll and proline content, higher germination rate and soluble sugar, lower lipid peroxidation	NA	[150]
Greenhouse	Drought	Higher soluble sugar and proline content, enhanced superoxide dismutase and peroxidase, improved cell membrane integrity, increased net photosynthesis, stomatal conductance, transpiration rate and root length	NA	[150]
Laboratory green house and field	Drought and salt	Increased proline and soluble sugar content, well developed roots, reduced leaf stomatal density, increase ROS scavenging enzymes	43% higher	[144]
Green house and field	Drought	Proline content and sugar increased, higher peroxidase activity, reduced loss of net photosynthetic rate, reduced lipid peroxidation, greater plant height, larger bolls	Yield increased	[141]

### CRISPR/Cas9 technology

In recent years, zinc finger nuclease (ZFN) and clustered regularly interspaced short palindromic repeat (CRISPR) has become a popular genome editing technology. This technology is highly important for creating genetically engineered plants as well as functional genomic study. These systems are associated with (Cas) 9 proteins and guide RNA (gRNA) is a rapidly developing genome editing technology which is effectively employed in many plants [151] such as rice [152], *Arabidopsis* [153], potato [154], watermelon [155], maize [156], tomato [157] and soybean [158]. Cas9 is composed of two nuclease domains in which the largely used one is resulted in from streptococcus pyogenes. The gRNA: is a synthetic 100 nucleotide (nt) RNA molecule. The first about 20 nt are the targeting site and the 3′end forms a hairpin structure that interacts with Cas9 protein [159]. A distinctive feature of CRISPR/Cas9 is that DNA cleavage sites are recognized through Watson–Crick base pairing [160] by three components: Cas9 protein, CRISPR-RNA (crRNA) and trans-activating crRNA (trancrRNA) [161]. The utilization of CRISPR/Cas9 system as a genome engineering tool came out when it was revealed that the target DNA sequence could be simply re-programmed by altering 20 nucleotides in the CRISPR-RNA [159]. In cotton, the application of CRISPR/Cas9 is still at its infancy. Most recently, multiple sites genome editing through CRISPR/Cas9 system in allotetraploid cotton by targeting arginase (GhARG), discosoma red fluorescent protein2 (DsRed2) and chloroplast development (GhCLA1) genes suggests that it is highly reliable and effective for cotton genome editing [162]. The ZFN is a laborious method because of complications in protein design, synthesis, and validation [163]. These issues were resolved with the exploration of CRISPR/Cas9 system, which saves time and cost, and is highly efficient [158]. The CRISPR/Cas9 system functions as an endonuclease and it induces double-strand breaks (DSB) at specific genome sites. In eukaryotic cells, such breaks are preferentially restored by the error-prone NHEJ (non-homologous 98 ends joining) pathway and often causes bases insertion, deletion resulting in gene function loss [164]. In plants, DSBs could be employed to knock-out genes [165], modify expression of gene by insert transgenes at a certain location via homologous recombination [166] or disrupting promoter sequences [167]. Considering the reported data about CRISPR technology; it will be a simple, time saving, cost effective and highly effective tool for plant gene expression, repression and genome editing. Thus, encouraging the application of genome editing tool to yield new alleles and engineer plants possessing required quality, agronomic traits and good drought tolerance. It is predictable that the possible applications of CRISPR/Cas9 in cotton genome editing are certain to be further established over time. In the future, advancements will continue to enhance their use from mutant generation to precise gene regulation at noncoding enhancer regions in cotton.

## Conclusions and future research directions

Sustainable crop production is a key goals of the current agricultural production systems. Drought is a major abiotic stress, limiting crop productivity in many parts of the world. Restricted water supply to cotton plants can impair normal physiological functioning through reduced nutrient supply and cellular toxicity. Therefore, improving crop performance under harsh environments has become an increasingly important issue. Despite major advances in genetic approaches, challenges to crop production in terms of genetic and environmental interactions for cotton lint production are still not fully understood. To date, limited data is available regarding the role of CRISPR/Cas9 technology, ascorbic acid, calcium, hydrogel application and the miRNAs under drought stress in cotton crops. There are still issues with the transgenic crops produced for inducing drought tolerance. Further, many transgenic plants have not been verified under natural conditions. Therefore, the performance under natural environment is still a question mark. Up to date information regarding drought-associated genes and their functions is poorly understood in cotton crops. Further research is inevitable to study these genes in response to drought stress under natural conditions as well as drought associated cotton protein kinases. Further research is needed for enhancing crop productivity under drought stress through interference (RNAi) technology. Cas9 will develop novel alleles, desirable agronomic and quality traits through engineer plants and will produce tolerance in crops plants against drought stress and or harsh environmental conditions.

## Abbreviations

CRISPR/Cas9: clustered regularly interspaced short palindromic repeat
P*n*: photosynthetic rate
Pro: proline
ABA: abscisic acid
SnRK2: sucrose non-fermenting 1-linked protein kinase 2
PP2C: protein phosphatase 2C
JA: jasmonic acid
JAZ: jasmonate-zim domain
JAI3/JAZ: jasmonate-insensitive/jasmonate-zim
MYC2: myelocytomatosis
MYC2: myelocytomatosis
ROI: active reactive oxygen intermediates
HO·: hydroxyl radical
O_2_^−^: superoxide anion radical
H_2_O_2_: hydrogen peroxide
^1^O_2_: singlet oxygen
AA: ascorbic acid
GSH: glutathione
GSH: glutathione
PEG: polyethylene glycol
CAT: catalyse
SOD: super oxide dismutase
POD: peroxidase
GST: glutathione S-transferase
CMC: carboxymethyl cellulose
SA: salicylic acid
GA_3_: gibberellic acid
miRNAs: microRNAs
ZFN: finger nuclease
gRNA: guide RNA
